# Management of adverse events in a Moroccan regional hospital: a state of art and perspectives

**DOI:** 10.11604/pamj.2024.47.69.41560

**Published:** 2024-02-19

**Authors:** Khadija Elwardi, Mohammed Bakkali, Amin Laglaoui

**Affiliations:** 1 Research Laboratory of Biotechnology and Biomolecular Engineering, Faculty of Sciences and Techniques of Tangier, Abdelmalek Essaadi University, Tangier, Morocco

**Keywords:** Adverse events, healthcare, Morocco, nosocomial infection, risk management

## Abstract

**Introduction:**

the risk management system is useful to identify, analyze, and reduce the risk occurrence of adverse events (AEs) in health services. This system suggests useful improvements to patients and to the whole institution and also contributes to the acquisition of a collective and organizational safety culture. This study presented a state of the art of the management of AEs identified in different services of a regional hospital in the north of Morocco.

**Methods:**

this is a retrospective cross-sectional exploratory study carried out from 2017 to 2019 using observations and semi-structured interviews, which were recorded, re-transcribed, and analyzed. Data was also collected from audit reports, results of investigations of the nosocomial infection control committee and the risk management commission, AEs declaration sheets, and meetings reports.

**Results:**

a number of 83 AEs were recorded, 10 of which were urgent. The reported events were related to care, infection risk, the drugs circuit, and medico-technical events. Two hundred cases of nosocomial infections were also recorded, of which 75 occurred in the intensive care unit and 35 in the maternity service. Surgical site infections were the most frequently reported complication. Adverse events were related to organizational failure, equipment problems, and errors related to professional practices.

**Conclusion:**

our findings may guide the improvement of the event management system in order to reduce the occurrence of future incidents. Thus, improving the risk management system requires setting up training strategies for staff on the importance of this system and its mode of operation.

## Introduction

The appearance of risks and adverse events (AEs) is a reality that is associated with the care and provided services, which can impact the patient, staff, and services. A poorly managed risk causes a problem in the performance of the institution, client dissatisfaction, and an inability to deal with emergency or exceptional situations.

Several AEs can be identified within hospital structures: patients error, foreign bodies after surgery, poor diagnoses, nosocomial infections (NIs), medication errors, etc. [1,2]. According to a report by the Directorate General of health in 2003 [3], iatrogenic events, including NIs, occurred in France in more than 10% of hospital stays. NIs are one of these iatrogenic events. Drug iatrogeny causes 25,000 deaths per year, which is four times more than road accidents, with a cost of 2.3 milliard euros for only avoidable events. Similarly, the national survey of severe AEs associated with care in France estimated that in 2009 severe AEs occurred between 5.1 and 7.3 for 1000 days of hospitalization (275,000 - 395,000 severe AEs/year) [4].

The management of these AEs becomes a major and priority issue. It aims to prevent the appearance of AEs and, in the case of their emergence, to identify, analyze the causes, and implement preventive and corrective improvement actions [5].

The development of AEs reporting systems has become necessary for all healthcare institutions [6]. The objective of this system is to develop a culture of risk management among healthcare professionals and to constitute a continuous learning tool for their services in order to improve patients care [1]. The systematized announcement of AEs and critical situations occurring within a hospital, particularly in clinical services, is essential for the good performance of this system. It is also interesting for professionals as it contributes to improving the care practices [7]. Collomp *et al*. suggested that essentially the potential risks should be rigorously identified and integrated among the indicators dedicated to management [8]. Once the quality unit receives the declaration, it initiates an investigation to better understand the event, to seek the organizational causes, and to identify the significant underlying actions. Different quality tools such as cause trees, consequence trees, and Ishikawa diagrams, can be used by the team to carry out well-structured reflective work [9]. Open communication and development of feedback on AEs in institutions should be undertaken in order to be part of a continuous improvement process [5,7]. It is noteworthy to mention that the evaluations of the risk management systems are rather disappointing [6]. Indeed, the declaration of AEs in hospital services is underused [10], and the frequency of AEs is underestimated, which leads to a failure to take these events into account by healthcare professionals (absence of establishment of preventive measures) [11].

The prevalence of NIs was estimated in Morocco in 2011 at 5.5% with 38.8% surgical site infections (SSI) [12]. In 2014, the project to set up quality and risk management systems was initiated by the Moroccan Ministry of Health in selected pilot site hospitals. Declared a priority by the general directorate, an institutional directive regarding the management of AEs has been introduced within a regional hospital in the North of Morocco. This was supported by the committee of nosocomial infection control and a local committee for processing and analyzing the declared AEs as well as a software for collecting declarations. This strong institutional will has permitted to promote a dynamic of continuous improvement, via an official structure and to extend it to the entire organization (from 2015). However, the risk management process set up within the hospital has been faced with organizational and operating obstacles.

The present study aims to evaluate the establishment of a system for reporting AEs within a regional hospital in North Morocco through a state-of-the-art on the management of these events reported by the professionals in the different services of the hospital (characteristics of AEs, number, criticality, type, causes and actions for improvement, etc.). We also discuss the encountered difficulties during the risk management process as well as suggestions and the possible prospects for its improvement within the hospital.

## Methods

**Study design and setting:** this is a retrospective exploratory study carried out in different services of a regional hospital in Northern Morocco from 2017 to 2019.

**Participants:** were included in this study the members (doctors/nurses) of the quality/risk management committee, the committee of nosocomial infection control, the operational hygiene team, the planification and care quality unit, the nursing care unit, the administrative affairs unit, the doctor affairs unit, and heads of services and nursing managers.

**Data sources/measurement:** we used semi-directive interview guides. The interviews were conducted individually with complete confidentiality of respondents. These interviews were completed by the collection of numerous observations and primary data. Analysis of the recorded and re-transcribed interviews was then conducted. Data and documents from the committee of nosocomial infection control and the “quality/risk” unit (audit reports and survey results, activity reports, declaration sheets, and reports of quality/risk management committee meetings) were also collected from 2017 to 2019.

**Variables:** the collected information were: a) number of AEs, NI declared and their type; b) distribution of these events by service; c) identification of patients (age, sex) for NI; d) consequences, causes/contributing factors of NI; e) improvement actions proposed by the committee of nosocomial infection control and the quality/risk management committee to control AEs.

**Ethical considerations:** this study was approved by the Ethics Committee for Biomedical Research of the Faculty of Medicine and Pharmacy of Rabat (CERB) (IORG0006594) and adhered to the Declaration of Helsinki. Informed verbal consent was obtained from all participants and was approved by the ethics committee.

## Results

### Descriptive data/outcome data and main results

**Adverse events (AEs) management circuit:** hospital manager and the quality/risk management committee implemented in 2016 an AE notification software available to service managers with secured password access. Information meetings about the operation and use of this informatic tool were planned and held with the head doctors of services, nursing managers, the manager of the nursing care unit, the administrative affairs unit, the doctor affairs unit, and other administrative and technical managers. This declaration and collection tool aims to improve the management and traceability of the declaration and to strengthen the adherence of institutions to this alert system. Based on the results of the interviews conducted with the members of the quality/risk management committee, it seems that this software was little used by the staff. The reporting was therefore mainly done by filling out standardized forms in paper format, which were then submitted to the manager of the planification and care quality unit.

**Figure 1:**
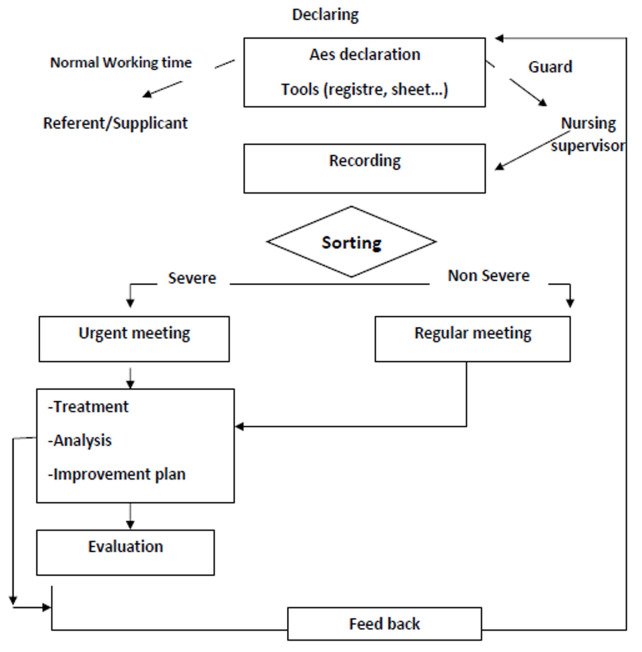
adverse events (AEs) management circuit adopted by the hospital quality/risk management committee

The quality/risk management committee collects and then analyzes the sheets of AEs occurring within a defined organizational framework. It also implements solutions to correct and prevent such events. Except for reports of urgent events, all reported AEs were processed monthly. Depending on the circumstances, the corrective measures, decided during an AE can be determined by the participation of the actors involved in the AE (Figure 1). Services managers were informed by the results of the summary of declared risks and AEs that occurred within their service, as well as by the monitoring and evaluation of improvement actions and by the reporting system.

**Figure 2:**
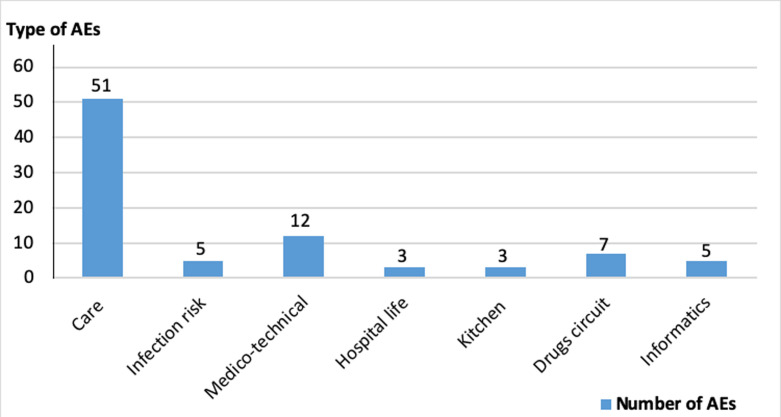
distribution of the declared adverse events (AEs) in different hospital services according to their types

**The AEs notified by the quality/risk management committee after the implementation of the reporting system:** in the first months after the deployment of the software, the quality/risk management committee received 83 AEs, of which 10 were urgent (3 AEs related to care, 2 AEs related to the infection risk, 2 medico-technical AEs, and 3 AEs related to the drugs circuit) (Figure 2). The recorded AEs were classified and assessed by the quality/risk management committee according to their criticality (high, medium, and low), their service of occurrence (operating room, surgical intensive care unit (ICU), etc.), and their types (AEs related to care, hospital life, drugs circuit, informatic, medico-technical) (Table 1).

**Table 1:** status of notifications processing by the risk management committee

Field of AEs	AEs	Criticality/initial risk level	Services	Proposed improvement actions
Care process	Hospitalized patient infection	Strong	Anesthesia-intensive care unit	Writing a protocol
				Meeting, training, and awareness of staff
				Purchase of single-use equipment
				Put single-use jars
				Disinfection
Care process	Dysfunction of the operating program (number of reported patients)	Medium	operating room	Checklist
				Decision of the medical staff (surgeons-ICU doctor-head doctor of service)
				Written procedure
				Simulation
Care process	Identity-vigilance non-respect of wearing a bracelet	Strong	All the services	Awareness and training of staff
				Organize the schedule
				Reminder of tasks
Hospital life	A significant number of reports of violence against staff	Strong	All the services	Awareness and training of staff
				Elaboration of action protocols
Outside	Vehicle access non-compliance with parking and traffic rules			Layout project
				Vehicle access rules
				Training of security guards
				Parking signage
Medico-technical (biomedical)	Risk of contamination by vacuum cleaners and filters	Medium		Verification of radiology rooms/vacuum cleaners and filters.
	Risk of burns by autoclave			Adding inverters.
	Lack of sites for hospital waste			Equipment inventory
	Non-separated of dirty and clean circuit for sterilization			Setup of central air treatment
				Study of the local crusher ventilation
Informatics	Technical problem related to the failure of computer equipment	Strong		Maintenance contract
	Accidents causing total or partial destruction of computer equipment: fire, flood, fall, food liquid, exposure to the sun...			Preventive maintenance
	Dysfunction due to electrical failures.			Renewal of the computer park
	Risk linked to virus attacks			Training of staff on good practices for using computer equipment/adding inverters
	Risk related to the security of hospital information			Working with file sharing and local mail server
				Block unwanted websites
				Adding passwords and defining access rights
				Take into account the issues of informatic system security in the policy of human resources management
Drugs circuit	Problem of drugs storage/delivery circuit by hospital	Strong		Create a cold room
	Failure of traceability of drug management			Build a repository that meets storage standards
	Transport between hospitals			Set up storage sheets in services
	The problem of shortage of emergency pharmaceutical products			Provide a vehicle for drugs transport between hospitals
	Loss of data			Plan a budget for emergency products
				Computerize the service
Fire	Cigarette in services	Strong		Regular staff training
				Alarm system (fire detector)
	Premises cluttered with easily flammable objects			Ensure the maintenance of firefighting equipment
				Evacuation protocol
	Blocked emergency exit			Release emergency exit
				Fire doors
	Improper use of electrical equipment			Have beacon lighting
				Cooperation with firefighters
Informatics	Network wiring diagram	Medium		Production of the plan
				Contact the former computer technician
				Take a survey
Maintenance	Electrical installation (power cut)	Strong		Visual display
				Inform the staff
				External electrical devices/conformity with traceability

Among the 51 declared events related to care, AEs included problems related to infection of hospitalized patients, dysfunction of the operating program (delay in scheduling patients), and problems related to identity vigilance (non-respect wristband wearing). The twelve medico-technical type incidents were related to the lack of sites for hospital waste, the problem of separating the dirty and clean circuit for sterilization, the risk of contamination by vacuum cleaners and filters, and also the risk of burn by autoclave. The seven AEs related to the circuit of drugs and medical devices resulted from problems of storage and drug delivery, failure of traceability of drug management, problem of shortage of emergency pharmaceutical products (lack of vital medicine ventolin in the hospital pharmacy and lack of central catheter for injection of adrenaline), need for some medical devices (need for filters and vents for dialysis machine) and problems of loss of data.

Three declarations of violence against staff were notified by the quality/risk management committee and also problems related to maintenance (power cut and breakdown of the electrogene group, which caused an arrest of dialysis machines during full dialysis session and loss of the dialysis blood circuit in five dialysis patients). As for informatics AEs, the declared technical problems were linked to failure of the informatics equipment, accidents that cause total or partial destruction of the informatics equipment (fire, flood, fall, exposure to the sun, etc.), dysfunction due to power outages, risk related to virus attacks and risk related to the security of hospital information.

During the same period, the quality/risk management committee recorded fire risks linked to organizational problems (premises cluttered with easily flammable objects, blocked emergency exits, improper use of electrical equipment and cigarettes in the services). The declared events were analyzed by the quality/risk management committee and corrective measures were suggested (Table 1, Figure 2). The 200 NIs cases reported by staff are not taken into consideration in this figure.

**Nosocomial infections (NIs) registered by the committee of nosocomial infection control of the hospital:** among 200 cases of NIs recorded, 75 declarations were from surgical intensive care, and 35 from maternity. The two services for men's surgery and traumatology represented 13.5% (27 cases each). Ten cases were reported for women's surgery and neurosurgery services each, and 14 cases were reported for children's surgery. In the operating room, only 2 cases of NIs were notified during the whole period. The most frequently recorded pathologies, which were of NIs origin were infections of the operating site and the complication of pulmonary infection for hospitalized patients in the ICU (Table 2). Of all registered patients (N=200), a large proportion of hospitalized patients (112) who developed NIs were aged between 30 and 45 years old. A total of 120/200 cases had lasting sequelae that were associated with prolonged hospitalization for almost all of the recorded cases (184/200). Among the 200 declared NIs, 8 (4%) led to death (Table 2).

**Table 2:** nosocomial infections declared by the committee for the fight against nosocomial infections at the hospital during 2018-2019

Inpatient service	Number of declared NIs (n)	Frequency (%)	Types or sites of NIs
Maternity	35	17.5	Surgical site infections
Children surgery	14	7	13 cases of osteomyelitis and 1 surgical site infection
Man surgery	27	13.5	22 urinary tract infections and 5 surgical site infections
Woman surgery	10	5	4 urinary tract infections and 6 surgical site infections
Intensive care	75	37.5	7 urinary tract infections related to the placement of the bladder catheter, 15 sepsis (passage of the germ through the venous pathway), and 53 pneumopathies
Operating room	2	1	Surgical site infections (internal infection)
Neurosurgery	10	5	Surgical site infections
Traumatology	27	13.5	20 surgical site infections and 7 osteomyelitis

Nis: Nosocomial infections

## Discussion

The management of AEs is one of the essential tools to develop a coherent approach to patient safety. The implementation of such a risk management system can help the organization to achieve its objectives, to determine the risks that could hinder this procedure, and thus allow the direction to identify profitable prospects [13]. Managing risks involves initially identifying and then dealing with incidents that occurred or may occur, and which may influence the health and safety of patients and staff. All staff should report such events.

Adverse events reporting systems have been implemented in some healthcare institutions in order to: facilitate the application of legal texts relating to the declaration of medical accidents, analyze the epidemiology of AEs by their classification based on their severity, allow the implementation of morbidity-mortality reviews [14] and prevent the emergence of risks by suggesting preventive improvement actions. Our study aimed to evaluate the implementation of an AE reporting system within the regional hospital of north Morocco by presenting a state-of-the-art management of AEs declared by professionals in the different services of the hospital. We also discussed the obstacles experienced during the implementation of the risk management approach as well as possible prospects for its improvement.

The study of declared AEs showed a predominance of incident declarations related to equipment, drugs, and also to care and organization. This observation was also found in several published studies [6,14,15], in which the declared AEs were incidents of care and medico-technical type, AEs linked to the circuit of drugs and medical devices. All of the declared and recorded AEs were systematically processed and analyzed by the commission of quality/risk management via meetings with the presence of nurses and senior doctors of the hospital services as well as the involved staff. Contributing factors are quickly identified by healthcare professionals. The systemic analysis was accompanied by corrective measures [14]. Such an analysis of AEs' criticality, taking into account the severity and the probability of AEs, could guide the declaration towards the most critical AEs [11].

Our participants preferred reporting NIs more than other AEs. This is in accordance with available data describing higher declarations of problems with a strong interaction with patients [6]. We showed that 200 cases of NIs with significant consequences (lasting sequelae, prolonged hospitalization, death, etc.) were recorded by the committee of nosocomial infection control. Of the 200 hospitalized patients who developed NIs, 112 were aged between 30 and 45 years old. A high rate of NIs was recorded in the intensive care unit (37.5%), followed by surgery services (31.5%), maternity (17.5%), and traumatology (13.5%). These results are in line with those found in French surveys, which described a prevalence of 22% of NIs in intensive care hospitalizations [16]. Invasive procedures, particularly surgical procedures, were responsible for a significant number of NIs during hospitalization [5].

The frequency and severity of healthcare-associated infections, which are particularly increased in intensive care units, were explained by the nature and diversity of the pathologies, comorbidities, and also by the excessive use of invasive techniques [17]. We describe in our study, diverse factors contributing to the occurrence of the reported NIs (according to the interviews conducted with the nurses and doctors heads of different services, the members of the committee of nosocomial infection control, the operational hygiene team, and also according to the data analysis from audit reports conducted by the committee of nosocomial infection control): non-respect of some care procedures and good practices; hand hygiene problem; bio-cleaning and the unsatisfactory disinfection (disinfection of surfaces, incubators, and medical equipment, etc.); the medical device sterilization services do not fulfill standards and waste sorting processes is not respected; non-respect of the operating room clothes; non-availability of material resources: (absence of some equipped water points and hydro-alcoholic solutions for hand hygiene, increasing need for hygiene products, etc.); insufficient human resources (non-functional operational hygiene team, shortage of staff, etc.); insufficient continuous training in hospital hygiene, hand washing, waste management, recommendations for good practices, etc.; room design problem (lack of isolation rooms, etc.).

These results are in line with other studies, which reported that AEs were linked to an organizational failure [5], a problem related to the equipment [5,14], errors of the non-medical or external care team, or linked to professional practice problems [14]. According to Carricaburu *et al*. in order to fight and prevent INs, it is necessary to emphasize several actions of quality improvement and risk management: communication of recommendations of good practice or protocols; training of healthcare professionals in “hospital hygiene”; strengthening the system surveillance and infection declaration, etc. [18].

According to the majority of interviewees, the number of AEs declared to the risk manager and the quality unit of the hospital does not reflect the real number of AEs. The number of these AEs is limited compared to the number of medical acts and care and the number of hospitalization days in the different hospital services. This finding is consistent with previously published data on AE reporting [14]. An AE reporting system aims to identify all situations of vulnerability of an institution [17]. However, a retrospective study conducted over 6 months in a Harvard hospital showed that the declaration rate was only 6% of the real rate of severe AEs [6].

It has been widely documented that the number of AEs detected by observation is higher than that reported by volunteer actors [19]. Another study regarding the declarations of AEs linked to medications demonstrated a number of severe AEs voluntarily declared, which is 10 times lower than those detected either by observers, records analysis, or by a computer system to detect medication errors [20]. All of the interviews revealed that under-declaration was an obstacle during the adoption of the risk management system within the hospital. Several factors led to this problem including, low level of staff involvement, poor perception of the importance of this system, lack of pedagogical support from superiors, fear of possible legal proceedings and affected reputation of practitioners, problem of application of corrective measures due to lack of resources, lack of feedback to declarants, etc.

The best result would be an increase in declarations proving a progressive mobilization of the actors [6]. Declaration and treatment of AEs are essential to build a culture of safety in institutions. This process needs deep collective analysis and morbidity-mortality meetings about safety themes and permits staff to think about the causes of incidents and improvement actions to manage risks [6,21,22]. The effectiveness of the risk management system largely depends on the existence of a safety culture. Thus, it is important to promote the circumstances for a culture of organizational learning based on the follow-up of incidents [6,7], open discussion about errors [23], and regular communication through the use of several communication channels to inform all the staff on the decisions taken (display in the service, emails, information meetings of medical and non-medical staff) [14]. The risk management culture also uses a constructive and participative non-punitive approach in a confident environment within the care services [7].

In order to improve the risk management system, it is necessary to set up awareness and training strategies for all staff and to develop and improve professional evaluation systems [24]. Organization of a risk management information system is also useful, especially as the hospital is within an accreditation process, which requires organizational procedures and a commitment to continuous improvement of quality and safety. Rojas *et al*. also suggested that hospitals should add questions on AEs in satisfaction surveys during patient discharge [23]. This could lead to better monitoring of patient safety [25,26]. Similarly, the AEs reporting system should be open to declarants external to the regional hospital center (doctors, caregivers, pharmacists or any person having contact with the organization, etc.) as well as for hospital collaborators [7].

## Conclusion

The implementation of the AE management approach, organized and supported by the general direction of the hospital, permits to initiate a dynamic of continuous improvement, prioritizing the quality of patient management and their safety and to promote the interprofessional collaboration. Our study presented a state of the art of all the reported AEs, their types, their causes, and the improvement actions proposed to avoid these AEs. We showed that AEs related to care, hospital life, the drugs circuit, and the medico-technical field were the most frequent. These incidents were mainly due to a failure of organization, equipment, and errors during professional practices. However, even being interesting, the impact of the feedback committee on the AEs declaration was not assessed in our study. A further study is needed to evaluate whether the implemented action plans will reduce the number of AEs. Our results revealed that the application of this system was faced several organizational obstacles. Thus, the common safety culture remains to be developed through the strengthening of the risk reporting system. Efficient communication on the declarations and the implemented corrective measures, could increase the number of the declared AEs. The direction of the hospital should also provide training and awareness sessions, for all the staff, on the importance of the AE management system and its mode of operation. The mobilization of healthcare professionals is therefore essential to advance the culture of risk management.

### What is known about this topic

- *The development of AEs reporting systems has become necessary for all healthcare institutions;*
- *The AEs reporting systems aim to develop the culture of risk management among healthcare professionals and to constitute a continuous learning tool in order to improve patients care*.

### What this study adds

- *Our study evaluates for the first time the implementation of an AE reporting system within the regional hospital of North Morocco;*
- *AEs related to care, hospital life, the drugs circuit, and the medico-technical field were the most frequent; these incidents were mainly due to a failure of organization, equipment, and errors during professional practices*.
